# Density of *Aedes aegypti* and *Aedes albopictus* and its association with number of residents and meteorological variables in the home environment of dengue endemic area, São Paulo, Brazil

**DOI:** 10.1186/s13071-015-0703-y

**Published:** 2015-02-19

**Authors:** Marianni de Moura Rodrigues, Gisela Rita Alvarenga Monteiro Marques, Lígia Leandro Nunes Serpa, Marylene de Brito Arduino, Júlio Cesar Voltolini, Gerson Laurindo Barbosa, Valmir Roberto Andrade, Virgília Luna Castor de Lima

**Affiliations:** Superintendência de Controle de Endemias, Praça Coronel Vitoriano, 23 Jardim Santa Clara, Centro, Taubaté, São Paulo CEP 12020-020 Brasil; Universidade de Taubaté, Avenida Tiradentes, 500, Bom Conselho, Taubaté, São Paulo CEP 12030-180 Brasil; Superintendência de Controle de Endemias, Rua Paula Souza, 166, Luz, São Paulo, São Paulo CEP 01027-000 Brasil; Superintendência de Controle de Endemias, Rua São Carlos, 546, Campinas, São Paulo CEP 13035-420 Brasil

**Keywords:** *Aedes aegypti*, Vector control, Culicidae, Dengue, Entomological indicator, Meteorological variables

## Abstract

**Background:**

Measure the populations of *Ae. aegypti* and *Ae. albopictus* adults according to sex and location inside or outside the residence, estimate *Ae. aegypti* female density per house and per resident, and test the association with abiotic factors.

**Methods:**

Adult mosquitoes were collected monthly with a hand net and portable electric catcher in the peridomiciliary and intradomiciliary premises of residences in an urban area with ongoing dengue transmission in the municipality of São Sebastião, Brazil, from February 2011 to February 2012.

**Results:**

Of the 1,320 specimens collected, 1,311 were *Ae. aegypti*, and nine were *Ae. albopictus*. A total of 653 male and 658 female of *Ae. aegypti* were recorded, of which 80% were intradomiciliary. The mean density of *Ae. aegypti* adult females was 1.60 females/house and 0.42 females/resident. There was an association between the number of females and the number of residents in both intradomiciliary and peridomiciliary premises (r^2^ = 0.92; p < 0.001 and r^2^ = 0.68; p < 0.001, respectively). There was an association between the number of females and the mean and total rainfall; the correlation was better in peridomiciliary premises (p = 0.00; r^2^ = 77%) than intradomiciliary premises in both cases (p = 0.01; r^2^ = 48%). Minimum temperature was associated in both environments, exhibiting the same coefficient of determination (p = 0.02; r^2^ = 40%). The low frequency of *Ae. albopictus* (seven females and two males) did not allow for detailed evaluation.

**Conclusions:**

*Ae. aegypti* is well established within the urban area studied, and the frequency of isolation is higher inside the houses. Female density was directly proportional to the number of residents in the houses. Our data show that human population density positively affects the number of *Ae. aegypti* females within the residence. Meteorological variables also affected mosquito populations. These data indicate a high probability of human-vector contact, increasing the possible transmission and spread of the DEN virus. Entomological indicators of adult females revealed important information complimenting what was obtained with traditional *Stegomyia* indices. This information should be a part of an interconnected data set for evaluating and controlling the vector.

## Background

Worldwide, *Aedes aegypti* is the primary vector of the virus that causes dengue, a disease that remains a serious public health problem in many tropical and subtropical countries. The control of this disease is directed towards the reduction of mosquito density in the urban environment, where it is predominantly found because it is a highly synanthropic species and has a blood-feeding preference for humans [1]. The endophilic and endophagic behaviour of *Ae. aegypti* adults, which can be found throughout the residential environment, is directly implicated in the successful transmission of the virus [1,2].

*Ae. albopictus*, a homologous species that is sympatrically distributed with *Ae. aegypti*, is epidemiologically important in transmitting the DEN virus throughout areas of Southeast Asia, and its vector competence is recognized in different areas, including Brazil [3].

*Ae. aegypti* surveillance is recommended by the WHO and adopted in many dengue-endemic countries to provide a quantifiable measurement of fluctuations in the magnitude and geographical distribution of the vector population [4]. The dengue control program employed in Brazil aims to collect larvae of this species in urban areas to estimate larval density. However, several authors have reported that larval indices do not meet the epidemic alert principle [5-7]. According to Focks [8], Barata *et al*. [9], Resende *et al*. [10], and Bowman *et al*. [11], the mosquito’s adult life cycle phase is most associated with transmission, and the female is the vector.

Several studies propose methods and indices to estimate the density of *Ae. aegypti* adult females [11-13]. These results have allowed the estimation of dengue’s critical thresholds and allowed for better understanding of its transmission dynamics, because monitoring the abundance of adult females highlights their distribution and density, which contribute to the development of control strategies.

Climatic factors are important in the dynamics of these insects and in the epidemiology of the diseases they transmit [14,15]. Mosquito life cycle parameters can be affected by abiotic factors, such as temperature, which affects egg viability; larval development; longevity; and adult dispersal, whereas rainfall affects the abundance and productivity of the breeding sites of these vectors [16]. In San Juan, Puerto Rico, the temporal dynamics of *Ae. aegypti* was positively associated with rainfall and temperature [17].

The adaptability of *Ae. aegypti* to more differentiated environments inhabited by humans reinforces the need for studies focusing on the behaviour of the adult female population within residences. Thus, it is important to evaluate the presence, distribution, and abundance of the adult forms of these vectors and their relationship with the human population; entomological indicators of this mosquito phase are essential parameters to better understand this vector’s ecology.

Thus, the present study aimed to investigate the abundance of the adult forms of *Ae. aegypti* and *Ae. albopictus* according to sex and location inside or outside houses in an urban area in a dengue-endemic municipality, to estimate the density of *Ae. aegypti* adult females per house and per resident, and to test their association with abiotic factors.

## Methods

This study was conducted from February 2011 to February 2012 in the São Sebastião municipality belonging to the northern coast of São Paulo State, Brazil. This municipality includes 73,833 inhabitants and is located at 45°21’00”W 23°21’20”S at an altitude of 15 m; it is 209 km from the capital. São Sebastião has a hot and humid climate with a mean annual temperature of approximately 20°C [18]. Its land area comprises 400 km^2^, of which approximately 300 km^2^ are covered by the Atlantic Forest biome.

The area studied in 2010 contained 13 neighbourhoods comprising 532 blocks consisting of 16,833 houses occupied by 40,116 inhabitants [18]. The selection of the blocks studied (n = 307) followed the method for random sampling of existing buildings within an urban area, according to the Manual on Entomological Surveillance for *Ae. aegypti* of the São Paulo State Secretary of Health (*Secretaria de Saúde do Estado de São Paulo*) for evaluating larval density [19]; residences were considered sampling units.

The municipality has been completely infested by *Ae. aegypti* and *Ae. albopictus* since the late 1990s [20]. In 2001, autochthonous dengue transmission began within the referenced area and continues to date [21].

Adults were collected once monthly, surveying a mean of 156 houses/month for five consecutive days. The collection method involved searching for adult forms in intradomiciliary and peridomiciliary premises of the houses using a hand net and aspirator, according to the method described by Nasci [22].

The fieldwork team comprised four operators; one pair surveyed intradomiciliary premises simultaneously as the other surveyed peridomiciliary premises. The mosquitoes were collected for 20 minutes per house in both environments at resting sites, such as furniture, clothes, curtains, etc. [12,23]. After collection in each house, the mosquitoes were transferred into appropriate tubes, and the samples were sent to the laboratory for identification [23].

In each block, work was initiated at the first house located on the northernmost corner. A sampling interval of three houses (survey one and skip two) was adopted, which corresponds to approximately 30% of the residences in each block; this procedure always occurred in a clockwise direction.

For analysing data, the STATISTICA program (version 7.0) was used with an alpha value of 0.05 after testing for normality (Shapiro-Wilk test) and homoscedasticity (Levene test). Two independent proportions tests were used to test the frequency of the adult individuals of each species. Simple linear regression analysis was used to establish the association between the number of *Ae. aegypti* adult females and abiotic variables (total rainfall, mean rainfall, mean temperature, maximum temperature, and minimum temperature). The following entomological indicators were calculated for *Ae. aegypti* females: adult house index (AHI) [5,12,23], adult female density/house (F/H) [12,23,24], and adult female density/resident (F/R) [23,25].

Daily meteorological data were obtained from the Centro Integrado de Informações Agrometeorológicas da Secretaria de Agricultura do Governo do Estado de São Paulo - CIIAGRO/SP.

## Results

A total of 2,036 houses were surveyed, and 1,320 adult mosquitoes were collected. Of these, 1,311 were *Ae. aegypti* (658 females and 653 males), and nine were *Ae. albopictus* (seven females and two males).

The number of *Ae. aegypti* specimens varied according to the residential environment throughout the study. Females and males of this species were more abundant in intradomiciliary premises (p = 0.00). However, no significant difference in sex was found in any residential environment (intradomiciliary premises, p = 0.53; peridomiciliary premises, p = 0.16) (Table 1).

**Table 1:** ***Aedes aegypti*** **and** ***Aedes albopictus*** **according to sex and residential environment**

**Location**	**Intradomiciliary**		**Peridomiciliary**		**Total**	
	**N**	**%**	**N**	**%**	**N**	**%**
*Aedes aegypti*						
Females	513	77.96	145	22.04	658	100.00
Males	484	74.12	169	25.88	653	100.00
Subtotal	997	76.05	314	23.95	1,311	100.00
*Aedes albopictus*						
Females	2	28.57	5	71.43	7	100.00
Males	2	100.00	0	0.00	2	100.00
Subtotal	4	44.44	5	55.56	9	100.00
Total	1,001	75.83	319	24.17	1,320	100.00

São Sebastião, northern coast of São Paulo, Brazil, February 2011 to February 2012.

Table 2 shows the monthly distribution of blocks and houses surveyed and those positive for *Ae. aegypti* adult females*.* There was a total of 174 (56.68%) positive blocks identified during the 13 months of study. The mean was 57.22%, ranging from 100% in March to 23% in September. Of the 2,036 houses, 367 contained *Ae. aegypti*, with a monthly mean of 17.76%, ranging from 47.83% in March to 6% in September.

**Table 2:** **Number of blocks and searched houses and positive for** ***Aedes aegypti*** **females**

**Month**	**Number of blocks**		**% block positivity**	**Number of houses**		**AHI**
	**Surveyed**	**Positive**		**Surveyed**	**Positive**	
Feb	23	16	69.57	170	41	24.12
Mar	19	19	100.00	159	76	47.80
Apr	21	19	90.48	170	41	24.12
May	20	12	60.00	164	26	15.85
Jun	23	11	47.83	128	12	9.38
Jul	25	10	40.00	155	12	7.74
Aug	19	6	31.58	159	12	7.55
Sep	23	5	21.74	150	9	6.00
Oct	26	11	42.31	150	17	11.33
Nov	28	18	64.29	152	32	21.05
Dec	31	18	58.06	166	32	19.28
Jan	26	16	61.54	149	32	21.48
Feb	23	13	56.52	164	25	15.24
Total	307	174	56.68	2,036	367	18.03
Mean	23.62	13.38	57.22	156.62	28.23	17.76
Standard error	0.98	1.31	6.04	3.16	5.03	3.07

São Sebastião, northern coast of São Paulo State, Brazil, February 2011 to February 2012.

The numbers of *Ae. aegypti* adult females per positive house and per house surveyed are shown by month in Table 3. The number of females/month varied in both cases, exhibiting mean densities of 0.32 and 1.60 females/house, respectively. The highest value was in March, and the lowest numbers were found in June and July.

**Table 3:** **Number of** ***Aedes aegypti*** **adult females and female density/house (F/H)**

**Month**	**No. of** ***Ae. aegypti*** **females**	**Number of houses**		**Female density/house (F/H)**	
		**Surveyed**	**Positive**	**Surveyed**	**Positive**
Feb	83	170	41	0.49	2.02
Mar	175	159	76	1.10	2.30
Apr	65	170	41	0.38	1.59
May	48	164	26	0.29	1.85
Jun	13	128	12	0.10	1.08
Jul	13	155	12	0.08	1.08
Aug	16	159	12	0.10	1.33
Sep	11	150	9	0.07	1.22
Oct	25	150	17	0.17	1.47
Nov	50	152	32	0.33	1.56
Dec	55	166	32	0.33	1.72
Jan	65	149	32	0.44	2.03
Feb	39	164	25	0.24	1.56
Total	658	2,036	367	4.12	20.81
Mean	50.62	156.62	28.23	0.32	1.60
Standard error	12.23	3.16	5.03	0.08	0.10

São Sebastião, northern coast of São Paulo State, Brazil, February 2011 to February 2012.

The number of females/resident (F/R) in the positive houses surveyed ranged from 0.27 in August to 0.64 in January and from 0.02 from July through September to 0.27 in March. The mean females/resident during the period studied was 0.09 in all houses surveyed and 0.42 in the positive houses (Table 4).

**Table 4:** **Monthly number of** ***Aedes aegypti*** **adult females/resident (F/R)**

**Month**	**N° of** ***Ae. aegypti*** **females**	**Total number of residents**		**N° of females/resident (F/R)**	
		**Surveyed houses**	**Positive houses**	**Surveyed houses**	**Positive houses**
Feb	83	598	138	0.14	0.60
Mar	175	638	278	0.27	0.63
Apr	65	748	196	0.09	0.33
May	48	579	102	0.08	0.47
Jun	13	467	34	0.03	0.38
Jul	13	541	46	0.02	0.28
Aug	16	679	60	0.02	0.27
Sep	11	499	36	0.02	0.31
Oct	25	697	63	0.04	0.40
Nov	50	537	140	0.09	0.36
Dec	55	560	143	0.10	0.38
Jan	65	464	101	0.14	0.64
Feb	39	542	89	0.07	0.44
Total	658	7,549	1,426	1.11	5.48
Mean	50.61	580.69	109.69	0.09	0.42
Standard error	12.23	24.47	19.45	0.02	0.04

São Sebastião, northern coast of São Paulo State, Brazil, February 2011 to February 2012.

**Figure 1:**
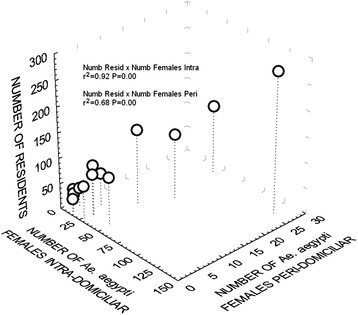
**Association between number of residents and number of** ***Ae. aegypti*** **females according to residential environment.** São Sebastião, northern coast of São Paulo State, Brazil, February 2011 to February 2012.

Figure 1 displays the association between the number of *Ae. aegypti* females captured in intradomiciliary and peridomiciliary premises and the number of residents per household. Our results show that this association was greater in intradomiciliary premises (r^2^ = 0.92).

**Figure 2:**
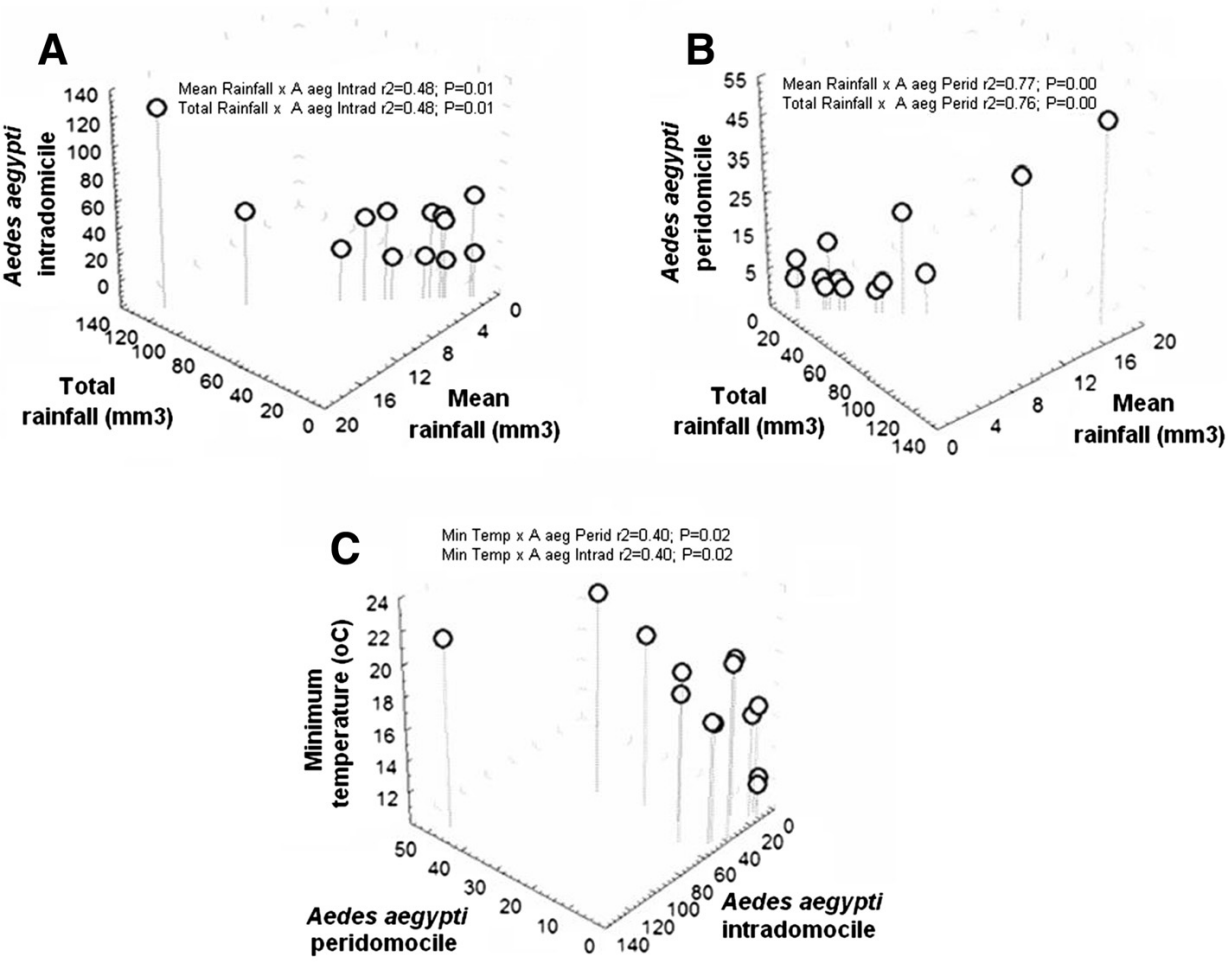
**Association between number of** ***Ae. aegypti*** **females and abiotic data. A**. Mean rain fall and total rain fall X *Ae. aegypti* in intradomicile. **B**. Mean rain fall and total rain fall X *Ae. aegypti* in peridomicile. **C**. Minimum temperature X *Ae. aegypti* in peridomicile and intradomicile.

The correlation analyses between the numbers of *Ae. aegypti* adult females and abiotic factors indicated correlations with mean rainfall, total rainfall, and the number of adult individuals in both intradomiciliary (p = 0.00; r^2^ = 77%) and peridomiciliary premises (p = 0.01; r^2^ = 48%) (Figure 2A and B). The minimum temperature revealed an association with the number of females (p = 0.02); however, the same coefficient of determination (r^2^ = 40%) was found for both environments (Figure 2C).

The total number of *Ae. albopictus* collected during the 13 months of study was very low (n = 9); five females were captured in peridomiciliary premises, and two females and two males were collected in intradomiciliary premises, thus preventing more detailed statistical analysis (Table 1).

## Discussion

In the present study, *Ae. aegypti* was more frequently encountered than *Ae. albopictus.* According to Forattini, [26] the urban environment favours *Ae. aegypti*, whereas rural and suburban areas favour *Ae. albopictus*. This environmental influence may be related to the ecology, biology, and behaviour of these species, which have distinct characteristics despite sharing other qualities.

The higher abundance of *Ae. aegypti* males and females in intradomiciliary premises corroborates reports in the scientific literature [2,27]. The presence of males in this environment is likely primarily related to the availability of shelter, because after emergence, the adult searches for sites with hiding places, where it can remain at rest during the period preceding the onset of activities [26].

The urban environment favours *Ae. aegypti,* which allows the females to feed on human blood within houses (anthropophily and endophagy). Additionally, these adults can oviposit and shelter themselves there (endophily). Several authors have reported this behaviour, which favours intradomiciliary premises for breeding grounds and the deposit of eggs [9,28,29]. Our data confirm previous findings on the bio-ecology of this species in an urban area, supporting the use of teams for the surveillance and control of adult forms of the vector in houses.

Entomological surveillance uses diverse indices to measure the levels of *Ae. aegypti* urban infestation*.* However, indices based on actual counts of the number of adult females are more accurate [8,10,30]*.* In the present study, the adult house index (AHI) showed *Ae. aegypti* female presence every month of the study in the blocks and residences surveyed, although different percentages were found, characterizing the distribution of the species in the urban environment. One explanation can be attributed to the aggregated distribution pattern of this *Stegomyia,* i.e., in finding individuals of the referenced species in a particular location, it is very likely to encounter others in surrounding areas [31-33].

The displacement of *Ae. aegypti* females seems to be associated with heterogeneity in the availability of blood and containers for laying eggs. According to Harrington et al., [34], the dispersal of these females is reduced in areas with physical and/or geographic barriers that limit their flight from 50 to 300 meters over their entire lives, which means that they would not often migrate beyond the block where they initiated their activities. When females find necessary resources near their point of origin, they most likely do not migrate large distances [6,26].

Gomes *et al*. [5] estimated house infestation in Foz do Iguaçu, Paraná, using the adult trap. They reported that *Ae. aegypti* was present in 9.6% of 6,661 residences surveyed. Although the same capturing method was not used, positivity also referred to the presence of females of this species. Nguyen *et al*. [12], in a cross-sectional descriptive study performed in Vietnam, reported that *Ae. aegypti* was present in 63% (n = 76) of the houses visited.

The female density values in the houses surveyed (F/H) in our study were close to those found by several authors. Chan [35] reported 0.2 females/house as a threshold for dengue occurrence in Singapore. Barata *et al.* [36] captured 0.4 females/house in São José do Rio Preto (SP), Brazil. Barata *et al.*, [9] in the municipality of Ocauçu and Uchoa (SP), captured 0.4 and 0.7 females/house, respectively, with resting boxes. Fávaro *et al.* [28], in studying density in two areas of Mirassol (SP), Brazil, reported that the mean number of females captured with aspirators ranged from 0.05 to 0.46 and from 0.08 to 0.62 at areas A and B, respectively. Nguyen *et al*. [12] used aspirators and found a density of 1.8 females/house in South Vietnam.

Females of this species are fast and persistent suckers capable of ingesting blood multiple times during a single gonotrophic cycle, a behaviour that increases the possibility of infecting and transmitting DEN viruses [26,37]. Dengue virus infection events were reported in areas with low densities of this mosquito [37,38]. Thus, performing studies that consider the population density of *Ae. aegypti* adult females transmitting dengue to show the quantitative relationship between vector indices and dengue cases could define a confidence level that could be used as a warning of impending dengue transmission; this may have been a limiting factor in this study.

Another important indicator of the risk of dengue transmission is related to human density and the number of *Ae. aegypti* females*.* This human-vector proximity has been reported by different authors. Chen* et al.* [39], in a study in Taiwan, showed a density of 0.07 females/person. Other authors in Colombia found 0.5 females/person [23]. In Trinidad, Chadee [40] found values of 0.6-0.7 pupae/person, but there was apparently no dengue transmission. Barrera [41], in Puerto Rico, corroborates the relationship between these variables, citing a mean of 0.99 ± 0.33 pupae/person. Basso *et al.,* [32] found 0.12 pupae/person in Paraguay. More recently, in a dengue transmission area of São Paulo State, in the region of São José do Rio Preto, 0.15 pupae/person were recorded [42].

In our study, the number of *Ae. aegypti* females captured in intradomiciliary and peridomiciliary premises was positively correlated with the number of residents, and the number was higher in intradomiciliary premises. These results indicate that this mosquito’s spatial distribution was affected by resident density/house; females of this mosquito maintain a close relationship with humans, and this proximity is favoured by places with shelter, among other aspects. These data suggest that the number of residents is a factor that attracts females to the residential environment, because the blood meal supply is larger. Getis *et al*. [43] and Harrington *et al.* [34] reported that people, instead of mosquitoes, may be the main mechanism of dengue virus transmission within and/or between communities. In a study in Hawaii using a spatiotemporal approach addressing the vector habitat and dengue, the authors concluded that human population density and urbanization are risk factors for dengue propagation [44]. Lin & Wen [45], in studying the relationships among mosquitoes, human density, and dengue incidence, found that a small number of *Ae. aegypti* females in a densely populated area may be sufficient to cause an outbreak of the disease, because a higher human density provides high vector contact rates.

Over time in the urban space, *Ae. aegypti* has adapted to its circumstances, because the environmental and social conditions are adequate for its proliferation and dispersal. Climatic conditions seem to promote increased geographical distribution of vectors, causing the expansion of some diseases [14].

In the present study, the association between the number of *Ae. aegypti* females and total rainfall or mean rainfall showed that rainfall affected the number of females collected in the residences; this effect was greater in the peridomiciliary premises. In regions with constant rainfall, such as the municipality studied here, extradomiciliary premises are not good places for adults to shelter, because they are more vulnerable to the weather. Thus, it can be deduced that when it rains, mosquitoes tend to find shelter in the vicinity of their breeding grounds; thus, they move toward more sheltered places. In a study performed in Puerto Rico [46], it was shown that *Ae. aegypti* is sensitive to changes in rainfall and that local climatic differences can contribute to dengue transmission.

In this study, minimum temperature was an important variable in determining the number of *Ae. aegypti* females captured in intradomiciliary and peridomiciliary premises. Câmara *et al*. [47], in a study performed in the city of the Rio de Janeiro, Brazil, showed an association between temperature and infestation by *Ae. aegypti*. They found that minimal temperatures recorded in the years of epidemics were higher, thus indicating that temperature is a limiting factor for virus multiplication in the vector organism. In the same city, Honório *et al*., [37] found that both temperature and rainfall were significantly related to *Ae. aegypti* abundance.

Regarding *Ae. albopictus*, although its occurrence was reduced, some aspects deserve to be mentioned to not overlook its potential role in transmitting arboviruses. Martins *et al*. [48] reported the first natural evidence of the vertical transmission of the dengue virus in populations of *Ae. aegypti* and *Ae. albopictus* collected in Fortaleza (CE), Brazil and opened a discussion about the epidemiological importance of this mechanism of viral transmission in the local scenario, especially concerning DEN virus management during inter-epidemic periods. In a study of the geographic expansion of these species, it was suggested that the abundance of the *Ae. albopictus* population decreased when *Ae. aegypti* was established [20].

Serpa *et al*. [49] showed the predominance of *Ae. aegypti* in São Sebastião (SP), the same municipality as the present study, whereas *Ae. albopictus* was found in very small numbers. The authors reveal the occurrence of an apparent habitat segregation process resulting from an urban gradient of occupation by these species.

## Conclusions

In conclusion, *Ae. aegypti* is well established in the urban area studied, presenting itself as permanent population with higher numbers inside houses. The female density was directly proportional to the number of people in the houses, from which one can infer a higher probability of the transmission and spread of DEN viruses at the local level. However, it is evident that the risk thresholds may be different at different sites and under different contexts. Similarly, rainfall and temperature were positively correlated with the number of females collected in the residences. The adult population of *Ae. aegypti* was easily sampled with the aspiration method throughout the study period. Our conclusion is that the entomological indicators of adult females must be part of an interconnected data set when evaluating and controlling this mosquito.

## Abbreviations

F/R: The number of females/resident
AHI: The adult house index
F/H: Adult female density/house
